# Optimization of Imaging Parameters for SPECT scans of [^99m^Tc]TRODAT-1 Using Taguchi Analysis

**DOI:** 10.1371/journal.pone.0113817

**Published:** 2015-03-19

**Authors:** Cheng-Kai Huang, Jay Wu, Kai-Yuan Cheng, Lung-Kwang Pan

**Affiliations:** 1 Department of Medical Imaging and Radiological Science, Central Taiwan University of Science and Technology, Taichung 406, Taiwan; 2 Department of Biomedical Imaging and Radiological Sciences, National Yang-Ming University, Taipei 112, Taiwan; Banner Alzheimer's Institute, UNITED STATES

## Abstract

Parkinson’s disease (PD) is a neurodegenerative disease characterized by progressive loss of dopaminergic neurons in the basal ganglia. Single photon emission computed tomography (SPECT) scans using [^99m^Tc]TRODAT-1 can image dopamine transporters and provide valuable diagnostic information of PD. In this study, we optimized the scanning parameters for [^99m^Tc]TRODAT-1/SPECT using the Taguchi analysis to improve image quality. SPECT scans were performed on forty-five healthy volunteers according to an *L*_9_ orthogonal array. Three parameters were considered, including the injection activity, uptake duration, and acquisition time per projection. The signal-to-noise ratio (SNR) was calculated from the striatum/occipital activity ratio as an image quality index. Ten healthy subjects and fifteen PD patients were used to verify the optimal parameters. The estimated optimal parameters were 962 MBq for [^99m^Tc]TRODAT-1 injection, 260 min for uptake duration, and 60 s/projection for data acquisition. The uptake duration and time per projection were the two dominant factors which had an *F*-value of 18.638 (38%) and 25.933 (53%), respectively. Strong cross interactions existed between the injection activity/uptake duration and injection activity/time per projection. Therefore, under the consideration of as low as reasonably achievable (ALARA) for radiation protection, we can decrease the injection activity to 740 MBq. The image quality remains almost the same for clinical applications.

## Introduction

Parkinson’s disease (PD) is a neurodegenerative disease characterized by progressive loss of dopaminergic neurons in the basal ganglia. Direct measurements of dopamine transporters (DAT) can indicate the severity of neuronal degeneration. Single photon emission computed tomography (SPECT) scans using [^99m^Tc]TRODAT-1 has been used to image DAT concentration and proven to be a valuable method to diagnose the early stage of PD [1] and other diseases [2–4]. The image quality of SPECT scans depends on the imaging parameters, which are determined empirically in the nuclear medicine department. Therefore, designing a dedicated scanning procedure for [^99m^Tc]TRODAT-1/SPECT is essential to promote diagnostic accuracy of dopamine-related diseases.

Various parametric settings existed for [^99m^Tc]TRODAT-1/SPECT scans. For the injection activity, 740 MBq (20 mCi) was given intravenously to predict the clinical response after treatment of the attention deficit hyperactivity disorder [5], while 925 MBq (25 mCi) was injected to make a differential diagnosis of vascular Parkinsonism and PD [6]. For the scanning time of SPECT, a total of 120 projections each with 20 s were acquired for imaging PD patients with social anxiety disorder [7], whereas 120 projections but each with 60 s were used for imaging patients with early-stage corticobasal degeneration [8]. For the uptake duration, 180 min was taken to investigate Japanese Encephalitis [9], whereas 240 min was selected to diagnose clinically unclear parkinsonian syndromes [10] and PD patients with depression [11].

The optimal imaging time for [^99m^Tc]TRODAT-1/SPECT has been investigated [12]. The result recommends that 240 min post-injection for clinical routine can achieve the highest caudate/occipital and putamen/occipital ratios. However, the image quality is influenced by multiple parameters interactively. Finding an optimal combination of the parameters and elucidating the relationships among them require extended investigation. In this study, we investigated the imaging parameters for [^99m^Tc]TRODAT-1/SPECT scans by the Taguchi analysis. Three parameters were considered, including the injection activity, uptake duration, and time per projection. The cross-interactions between these parameters were evaluated. The final goal was to develop a dedicated acquisition procedure for [^99m^Tc]TRODAT-1/SPECT scans.

## Materials and Methods

### Taguchi analysis and robust design

The Taguchi method is a kind of fractional factorial testing, which allows us to simultaneously examine several key elements in one study. It has become a popular tool for designing a high-quality system and has been applied in various research fields [13–16]. With a properly designed orthogonal array and the use of *F*-test, the Taguchi method can evaluate the sensitivity and significance of different imaging parameters, providing a more efficient way to establish scanning procedures used in nuclear medicine departments. Recently, it has been used to optimize the image quality of Ga-67-citrate gamma camera scanning [17]. Detailed descriptions of the Taguchi method and robust design can be found in other literatures [18–20].

### Orthogonal array

Three design parameters, including the injection activity, uptake duration, and acquisition time per projection, were considered with three various levels of each for [99mTc]TRODAT-1/SPECT scans. A total of 27 (3×3×3) different combinations are shown in Table 1. According to Taguchi’s recommendation [18], an *L*_9_ orthogonal array was used to rearrange the levels of parameters (Table 2).

**Table 1:** Three design parameters including the injection activity, uptake duration, and time per projection each with three different levels.

Symbol	Design parameter	Unit	Level 1	Level 2	Level 3
A	Injection activity	MBq (mCi)	740 (20)	851 (23)	962 (26)
B	Uptake duration	min	180	220	260
C	Time per projection	s/projection	40	50	60

**Table 2:** Parametric layout in an *L*_9_ orthogonal array of nine groups.

Group	Level A	Level B	Level C
1	1	1	1
2	1	2	2
3	1	3	3
4	2	1	2
5	2	2	3
6	2	3	1
7	3	1	3
8	3	2	1
9	3	3	2

The number in each column indicates various levels for the specific parameters.

### Human subjects

A total of forty-five healthy volunteers (age of 24–57 years old and mean age of 41.3±12.0 years), who were reviewed by a neurologist to exclude those with psychiatric diagnoses/medications, neurological diseases, and a history of movement disorders [21], were recruited. All protocols were approved by the Institutional Review Board of Show Chwan Memorial Hospital for clinical research, and all volunteers participated in this study gave written informed consent.

### SPECT scans

Nine groups were formed in the orthogonal array (Table 2). Each group contained five randomly assigned subjects; thus, a total of 45 normal participants were included, and the corresponding level of imaging parameters was applied according to Table 1. A dual head gamma camera (Sophia DXT-XLi, GE/SMV, Versailles, France) was used and a total of 60 projection angles were acquired over 180° for each detector head. The image was reconstructed into a 128 × 128 matrix with a pixel size of 2.11 × 2.11 mm^2^ using the filtered back-projection (FBP) algorithm with a Metz filter (order of 3.5 and cutoff of 7 cm^−1^). The slice thickness was 2.9 mm. Subsequently, photon attenuation correction was performed using the Chang’s first-order method with a broad-beam attenuation coefficient of 0.12 cm^−1^ [22, 23].

**Fig 1:**
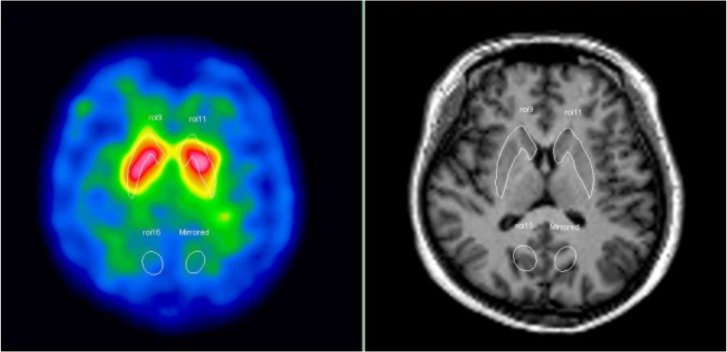
ROIs drawn for the striatum (upper) and occipital (lower) areas on the composite image.

The reconstructed image with the highest signal in the basal ganglia was extracted and summed together with its two adjacent slices as a single composite image. Several regions of interest (ROIs) were drawn by a neuroradiologist in the striatum and occipital areas at each hemisphere on the composite image, based on reference of the corresponding T1-weighted MR images (Fig. 1) [24]. The striatum/occipital (S/O) activity ratio of the subject was taken as the specific to non-specific uptake ratio, which is expected to be the higher the better owing to accumulation of [^99m^Tc]TRODAT-1 in the striatum. The signal-to-noise ratio (SNR) for each group of the orthogonal array was analyzed as follows:

$$\eta =-10\log\left[\frac{1}{n}\sum_{i=1}^{n}y_{i}^{-2}\right],$$ (1)

where *η* is the SNR in dB, *y*_*i*_ is the S/O activity ratio of the *i*th subject, *n* is the total number of subjects in each group (*n* = 5). A larger SNR is considered preferable. Namely, the optimal level of parameters is that with the highest *η*. The average SNRs for different levels of parameters were further calculated to evaluate the sensitivity of each parameter.

### Analysis of variance, ANOVA

ANOVA was used to provide a measurement of confidence and to determine which parameters were statistically significant. Variance was decomposed; the sum of squared deviation for a specific parameter (*S*_*x*_) and the sum of squared error (*S*_*E*_) were calculated as follows:

$$S_{x}=\frac{n\times r}{L}\sum_{k=1}^{L}{\left({\bar{y}}_{xk}-\bar{y}\right)}^{2},$$ (2)

$$S_{E}=\sum_{i=1}^{n}S_{i}^{2}\times (r-1),$$ (3)

where $\bar{y}$ is the mean S/O ratio of all subjects, ${\bar{y}}_{xk}$ is the mean S/O ratio for the *k*th level of parameter *x*, *n* is the number of groups (*n* = 9), *r* is the number of trials in each group (*r* = 5), *L* is the number of levels for each parameter (*L* = 3), and *S*_*i*_ is the standard deviation for the *i*th group. Subsequently, the *F*-test [25] was performed as follows:

$$F_{x}=\frac{S_{x}/f_{x}}{S_{E}},$$ (4)

where *F*_*x*_ is the *F*-value of the specific parameter *x*, *f*_*x*_ is the degree of freedom, and *S*_*x*_/*f*_*x*_ is the variance. The *F*-test is an auxiliary tool to examine whether the factors are dominant in the system. If the confidence level of the *F*-value is greater than 99%, the imaging parameter is considered statistically significant. Additionally, the larger the *F*-value, the more dominant the parameter.

The isopreference curve, defined as the points on the curve with the same image quality, was further analyzed by fixing the level of one parameter and changing the level of the other two parameters. The corresponding SNR was normalized by the average SNR of the level of the fixed parameter to eliminate its effect. The relationship between parameters can be revealed.

### Verification

For further verification, SPECT scans were performed on ten additional healthy subjects (age of 45–64 years old with average age of 52.9 years) and fifteen PD patients (age of 45–76 years old with average age of 60.7 years) using the predicted optimal parametric setting found by the Taguchi analysis and the conventional setting previously used in our hospital. For the conventional setting, the injection activity of 851 MBq, uptake duration of 220 min, and scanning time of 50 s/projection were applied. The average S/O ratios of healthy subjects and PD patients were estimated and compared for statistically significant using the unpaired *t*-test.

## Results

### Interpretation of SNR

The levels of parameters used for the *L*_9_ orthogonal array are summarized in Table 3. Table 4 lists the estimated S/O ratios for each subject and the corresponding SNRs for each group (see also S1 Table). Group 1 had the lowest SNR of 6.57 dB among all the groups. When the levels of uptake duration and time per projection rose from 1 to 3 (group 3), the SNR increased 53% to a maximum of 10.08 dB. This finding suggests that the uptake duration and scanning time per projection have a positive impact on the image quality.

**Table 3:** Nine study groups in the *L*_9_ orthogonal array with three different levels of parameters according to the Taguchi’s recommendation.

Group	Injection activity (MBq)	Uptake duration (min)	Time per projection(s/projection)
1	740	180	40
2	740	220	50
3	740	260	60
4	851	180	50
5	851	220	60
6	851	260	40
7	962	180	60
8	962	220	40
9	962	260	50

**Table 4:** Estimated S/O ratios for the normal subjects and corresponding SNRs for the nine groups.

Group	*y*_1_	*y*_2_	*y*_3_	*y*_4_	*y*_5_	Average S/O ratio	SNR, *η* (dB)
1	2.51	2.12	2.21	2.01	1.93	2.16 ± 0.22	6.57
2	2.41	2.47	2.36	2.25	2.47	2.39 ± 0.09	7.56
3	3.02	3.16	3.22	3.42	3.18	3.20 ± 0.14	10.08
4	2.46	2.53	2.63	2.57	2.48	2.53 ± 0.07	8.07
5	2.86	2.67	2.77	2.89	2.98	2.83 ± 0.12	9.03
6	2.32	2.74	3.23	2.91	2.89	2.82 ± 0.33	8.84
7	2.86	2.87	2.74	2.97	3.65	3.02 ± 0.36	9.47
8	2.34	2.65	2.63	2.29	2.45	2.47 ± 0.16	7.82
9	2.87	2.75	3.07	2.93	2.95	2.91 ± 0.12	9.27

The SNR is expected to be the higher the better.

*y*_*i*_ is the S/O ratio of the *i*th subject in each group.

**Fig 2:**
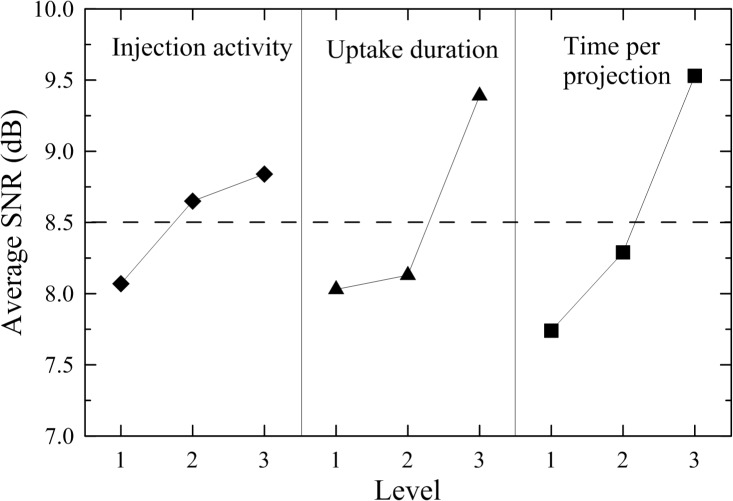
The main effect graph for different levels of parameters. The injection activity was less sensitive to levels than the uptake duration and time per projection.

The average SNRs of the three levels for the injection activity, uptake duration, and time per projection are presented in Table 5 (see also S2 Table) and the main effect graph in Fig. 2. The average SNR for the time per projection significantly increased 23% from level 1 to level 3, while it increased approximately 17% for the uptake duration. Relatively, the average SNR for the injection activity had the slowest slope among the three parameters and the improvement was less than 10%, implying that the injection activity is insensitive to SNR.

**Table 5:** Average SNRs of the three levels for the injection activity, uptake duration, and time per projection.

Parameter	Level 1 (dB)	Level 2 (dB)	Level 3 (dB)
Injection activity	8.07	8.65	8.85
Uptake duration	8.03	8.13	9.40
Time per projection	7.74	8.30	9.53

### Cross interactions between parameters

**Fig 3:**
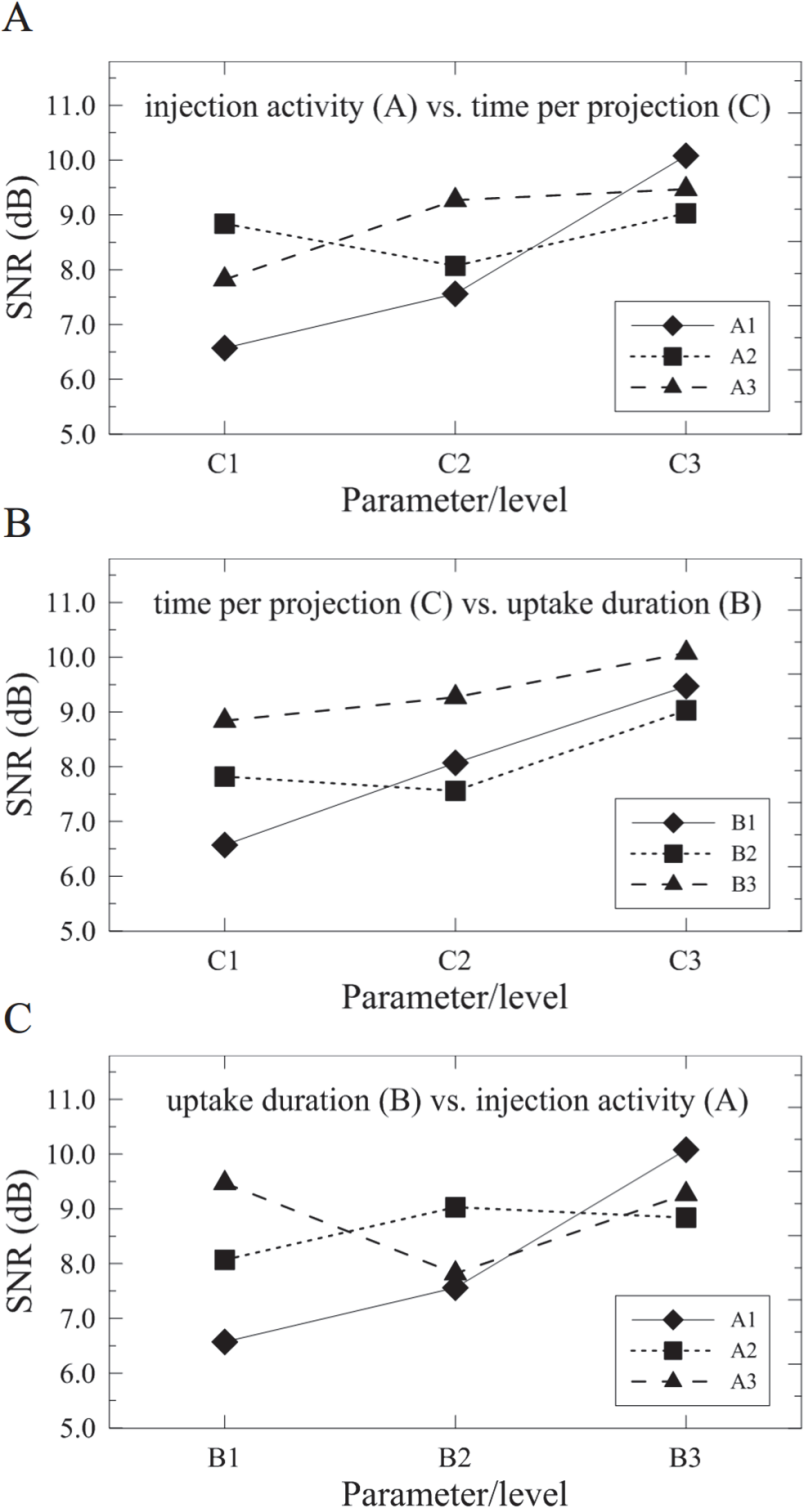
Major cross interactions between (A) injection activity and time per projection, (B) time per projection and uptake duration, and (C) uptake duration and injection activity.

As clearly depicted in Fig. 2, the optimal setting for acquiring the highest SNR should be set as 962 MBq (level 3) for the injection activity, 260 min (level 3) for the uptake duration, and 60 s/projection (level 3) for the scanning time. However, to further inspect the cross interactions between parameters as indicated in Fig. 3, the injection activity had strong cross interactions to either the time per projection (Fig. 3A) or uptake duration (Fig. 3C). Therefore, it may only hold the injection activity in the minimum amount to maintain the maximum SNR. Moreover, the cross interaction between the time per projection and uptake duration (Fig. 3B) depicted a weak correlation and agreed well to the calculated results as implied in Fig. 2. Namely, both parameters in level 3 achieve the maximum SNR. Therefore, by concerning the multiple cross interactions between parameters, another possible candidate for parametric setting becomes as the same as indicated in group 3.

### ANOVA and *F*-test

Table 6 shows the *F*-test results as well as the confidence levels for the three parameters. If the confidence level exceeds 99%, the parameter is considered statistically significant [25]. The uptake duration and time per projection were the two significant factors in which the latter one dominated due to its largest *F*-value of 25.933. The second priority was the uptake duration which contributed 38.27% of the total *F*-value. The injection activity, on the other hand, was a minor factor which occupied only approximately 8.48% of the total *F*-value and was not statistically significant.

**Table 6:** *F*-test results and confidence levels for the three parameters.

Design parameter	*S*_*x*_	*f*_*x*_	*V*_*x*_	*F*-value	Confidence level
Injection activity	0.372	2	0.186	4.132	97.28%
Uptake duration	1.678	2	0.839	18.638	100.00%
Time per projection	2.335	2	1.168	25.933	100.00%

The uptake duration and time per projection were the two significant factors since their confidence levels were over 99%.

*S*_*x*_ is the sum of squared deviation.

*f*_*x*_ is the degree of freedom.

*V*_*x*_ is the variance.

### Verification

**Fig 4:**
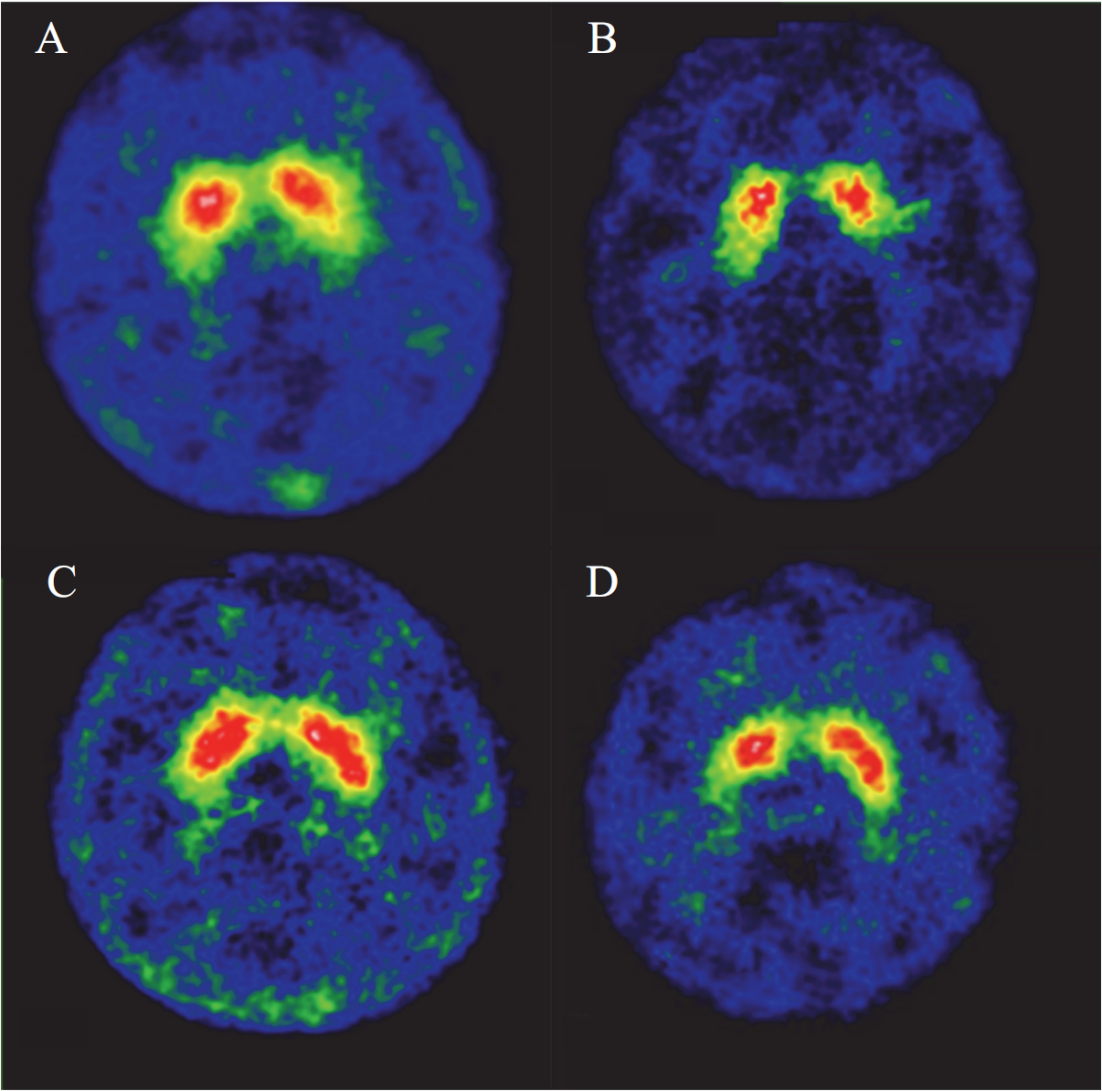
[^99m^Tc]TRODAT-1/SPECT images using the conventional setting for (A) a 34-year-old healthy female and (B) a 48-year-old male with PD, and using the optimal setting for (C) a 56-year-old healthy male and (D) a 45-year-old female with early PD.

Fig. 4 illustrates some [^99m^Tc]TRODAT-1/SPECT images of the subjects using the conventional setting and the optimal setting. Table 7 indicates the average S/O ratios and SNRs for the normal subjects and PD patients for verification (see also S3 Table). The data from the original group 3 in Table 4 are also listed for comparison, since it fulfills the primary Taguchi’s suggestion on the basis of considering cross interactions between parameters. For the healthy subjects, the SNR of the optimal setting was 25% higher than that of the conventional setting, and the difference of the average S/O ratios was statistically significant (*P*<0.01). The optimal setting had a slightly higher average S/O ratio than the result of the group 3 setting. However, there was no significant difference (*P* = 0.955). For the PD patients, the differences of the average S/O ratios between the conventional/optimal settings and the conventional/group-3 settings were not statistically significant. Additionally, the average S/O ratios between the healthy subjects and PD patients all showed significant differences (*P*<0.01) under these three parameter settings. These findings confirm that 962 MBq, 260 min, and 60 s/projection are the best combination for [^99m^Tc]TRODAT-1/SPECT scans. Additionally, the group 3 setting can achieve fine image quality comparable to the optimal setting.

**Table 7:** Comparison of the average S/O ratios and SNRs between the normal subjects and PD patients using the conventional setting, predicted optimal setting, and original group-3 setting.

Parameter	Conventional setting	Optimal setting	Original group 3
Injection activity (MBq)	851	962	740
Uptake duration (min)	220	260	260
Time per projection (s/proj.)	50	60	60
Normal subjects			
SNR (dB)	8.12	10.16	10.08
Average S/O ratio	2.55 ± 0.12	3.23 ± 0.17*	3.20 ± 0.14^†^
PD patients			
SNR (dB)	1.16	1.53	1.31
Average S/O ratio	1.19±0.23	1.25±0.24^‡^	1.24 ± 0.26^§^

*significant difference from the conventional setting (*P*<0.01)

^†^no significant difference from the optimal setting (*P* = 0.955)

^‡^no statistical significance from the conventional setting (*P* = 0.705)

^§^no statistical significance from the conventional setting (*P* = 0.719)

**Fig 5:**
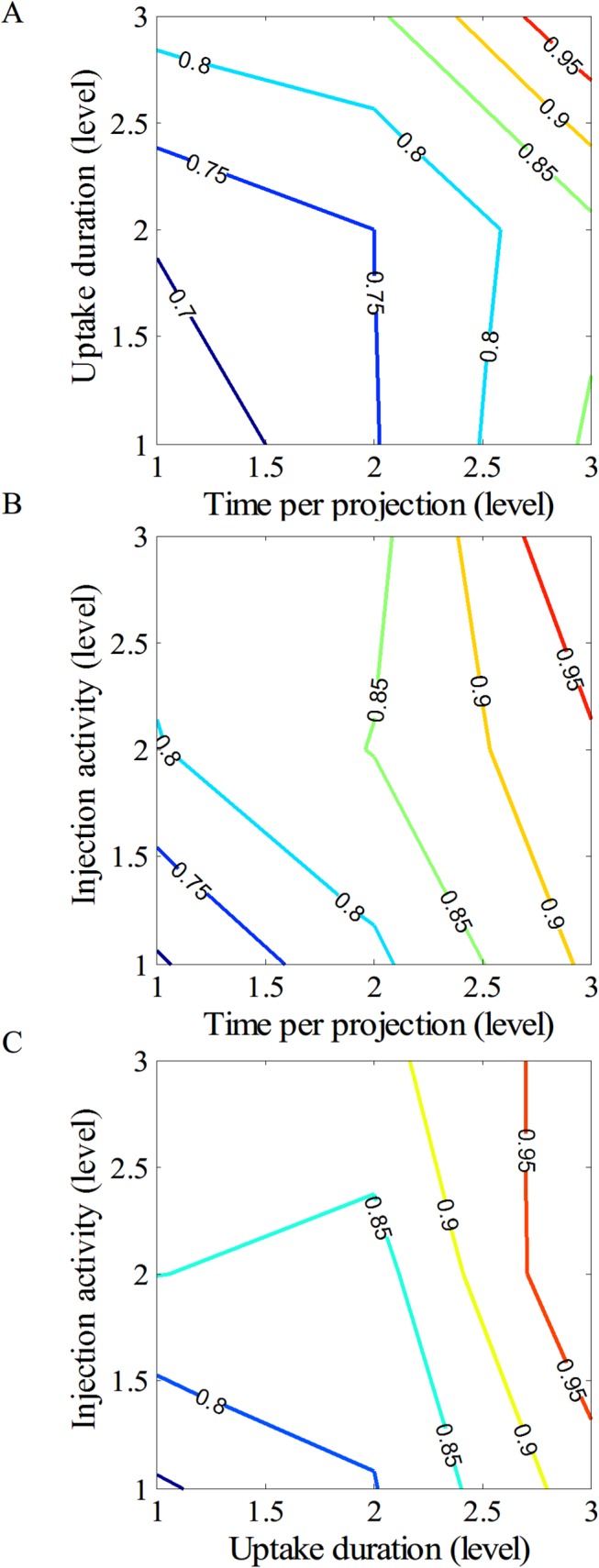
Isopreference curves through the level space for (A) uptake duration and time per projection, (B) injection activity and time per projection, and (C) injection activity and uptake duration. The number on the curve indicates the percentage of image quality.

By fixing one of the parameters, isopreference curves through the level space defined by the other two parameters are drawn in Fig. 5. Points lying on the curve correspond to the levels achieving the same image quality. The isopreference curves shifted upper right to level 3. With this flexibility, the scanning protocol can be adjusted based on the patient condition, such as the ability to remain still for a longer acquisition time or the reduction of radiation doses by injecting less radioactivity and adjusting the other two variables.

## Discussion

The scanning time per projection is the most dominant parameter in [^99m^Tc]TRODAT-1/SPECT scans. This is mainly because the statistical variation in counts depends on the number of photons interacting with scintillation detectors. The quantum noise, the major source of image degradation in nuclear medicine, can be decreased by increasing the acquisition time. However, we should not prolong the total scanning time to more than 60 min [26]. Patients may feel uncomfortable and undesired motion blurring could jeopardize the image quality [27]. Motion during data acquisition can also result in an underestimation of DAT concentration, leading to quantitative errors and misdiagnosis [28].

The uptake duration is another dominant factor which can provide a superior and stable target to non-target ratio. The uptake duration applied in numerous studies ranged from 180 to 240 min for [^99m^Tc]TRODAT-1/SPECT scans [29,30]. Based on our results, a longer delay time of 260 min is suggested for clinical practice. This recommendation is slightly longer than the imaging time investigated from other study [12]. Further prolonged uptake duration is not recommended due to additional biological and physical decay of radiotracers in the stratum [31].

The amount of [^99m^Tc]TRODAT-1 injected for SPECT scans usually ranges from 740 to 962 MBq. Our results indicated that 962 MBq can achieve a slightly higher SNR than 740 and 851 MBq, but the differences were not statistically significant. Obviously, increasing the injection activity is straightforward to gain more photon counts. However, dead-time loses of the counting system may become severe and compromise the benefit of increasing activity. Another drawback is that the internal organ dose of patients increases as the injection activity increases. The dose-limiting organ, liver, receives approximately 45.2 mGy (0.047 mGy/MBq) and the effective dose achieves 11.6 mSv (0.012 mSv/MBq) for 962-MBq injection [32]. Comparing with other ^99m^Tc-labeled compounds, the effective dose for [^99m^Tc]TRODAT-1 is generally higher [33]. Therefore, a tradeoff should be made between the additional radiation risk and image quality, since the injection activity is a minor parameter in terms of SNR. We suggest that the minimum amount of 740 MBq, which still holds the Taguchi’s recommendation on the basis of considering cross interactions, should be used according to the concept of as low as reasonably achievable (ALARA) in radiation protection.

In medical research, single factor experiments are often conducted. Although considering only one variable at a time is simple and direct, the optimal results may not be revealed due to that the effect of one variable depends on the values of other variables. These cross interactions frequently occur in real-life situations. The Taguchi analysis is a fractional factorial testing which can significantly reduce sample sizes and examine several key parameters at the same time. Through the system design, parameter design, tolerance design, and verification phases, we can obtain useful information about the problem domain. More importantly, the cross-interaction between parameters should be evaluated carefully before giving suggestions to prevent potential bias of results.

The average age of the normal participants is younger than that of the PD cohort. This is because we recruited younger volunteers for the optimization study to prevent additional factors affecting DAT function with aging. In the verification study, the average age of the normal subjects is 52.9 years (age of 45–64 years old), which is higher than that of the subjects in the parameter optimization. The optimized parameters can still enhance the SNR. In this study, the average age of the PD cohort is 60.7 (age of 45–76 years old). We did not examine the effect of optimized scanning parameters on early onset PD patients, which is the limitation of this study.

In addition to the three major parameters examined in this study, other parameters could affect the image quality as well. Future work will focus on using statistical reconstruction algorithms, such as the Ordered Subset Expectation-Maximization (OSEM) and the Maximum a Posteriori (MAP). The iterative methods can provide outstanding spatial resolution between the caudate and putamen, and may gradually replace the traditional FBP algorithm for quantification [34]. The impact on the optimized scanning procedure should be further investigated.

## Conclusion

In this study, we applied the Taguchi analysis to investigate the optimal imaging parameters for [^99m^Tc]TRODAT-1/SPECT scans. Our analysis indicates that 962 MBq for the injection activity, 260 min for the uptake duration, and 60 s/projection for the acquisition are the best choice in terms of image quality. Since the injection activity is a minor factor, 740 MBq should be used to reduce the internal radiation dose according to ALARA. The optimal imaging parameters could be applied to clinical practice to elevate the diagnostic accuracy of PD and other dopamine-related disorders.

## Supporting Information

S1 Table The numerical data set of the 45 normal volunteers extracted and analyzed from their image data. (DOCX)

S2 Table The calculation of average SNRs for different levels. (DOCX)

S3 Table The raw data of the ten normal subjects and 15 PD patients to calculate the average S/O ratios and SNRs. (DOCX)
