# Supplementary material for: Optimization of Imaging Parameters for SPECT scans of [99mTc]TRODAT-1 Using Taguchi Analysis
Source: PLoS One. 2015 Mar 19;10(3):e0113817. doi: 10.1371/journal.pone.0113817 (PMC4366084; doi:10.1371/journal.pone.0113817)
Supplement: S1 Table — (DOCX) [file pone.0113817.s001.docx]

**Table S1. The numerical data set of the 45 normal volunteers extracted and analyzed from their image data.**

| **Group** | ***y*_1_** | ***y*_2_** | ***y*_3_** | ***y*_4_** | ***y*_5_** |
| --- | --- | --- | --- | --- | --- |
| 1 | S:257.9  O: 73.4 | S:148.2 O: 47.5 | S: 137.2 O: 42.7 | S: 188.4 O: 62.5 | S: 165.5  O: 56.4 |
| 2 | S: 214.8 O: 62.9 | S: 281.9 O: 81.2 | S: 175.7 O: 52.3 | S: 176.7 O: 54.3 | S: 225.7  O: 65.1 |
| 3 | S: 172.1 O: 42.8 | S: 169.9 O: 40.8 | S: 175.1 O: 41.5 | S: 143.8 O: 32.5 | S: 190.8  O: 45.7 |
| 4 | S: 157.9 O: 45.6 | S: 193.9 O: 54.9 | S: 151.1 O: 41.6 | S: 209.7 O: 58.7 | S: 255.4  O: 73.3 |
| 5 | S: 224.9 O: 58.2 | S: 244.9 O: 66.8 | S: 185.7 O: 49.3 | S: 193.9 O: 49.9 | S: 271.9  O: 68.3 |
| 6 | S: 214.9 O: 64.8 | S: 206.8 O: 55.3 | S: 266.8 O: 63.1 | S: 272.1 O: 69.6 | S: 192.4  O: 49.4 |
| 7 | S: 183.1 O: 47.4 | S: 255.9 O: 66.2 | S: 211.9 O: 56.6 | S: 250.8 O: 63.1 | S: 199.6  O: 42.9 |
| 8 | S: 165.4 O: 49.5 | S: 250.1 O: 68.5 | S: 194.1 O: 53.5 | S: 210.9 O: 64.1 | S: 150.2 O: 43.5 |
| 9 | S: 248.1 O: 64.1 | S: 197.1 O: 52.5 | S: 236.4 O: 58.1 | S: 156.4 O: 39.8 | S: 178.8 O: 45.3 |

S: mean counts per pixel in the whole striatum

O: mean counts per pixel in the occipital cortex

The S/O ratio, average S/O ratio, and SNR (equation 1) in Table 4 can be calculated through these data.
