# Supplementary material for: Optimization of Imaging Parameters for SPECT scans of [99mTc]TRODAT-1 Using Taguchi Analysis
Source: PLoS One. 2015 Mar 19;10(3):e0113817. doi: 10.1371/journal.pone.0113817 (PMC4366084; doi:10.1371/journal.pone.0113817)
Supplement: S2 Table — (DOCX) [file pone.0113817.s002.docx]

**Table S2. The calculation of average SNRs for different levels.**

| Injection activity | Group | Average SNR |
| --- | --- | --- |
| Level 1 | 1,2,3 | (6.57+7.56+10.08)/3 = 8.07 |
| Level 2 | 4,5,6 | (8.07+9.03+8.84)/3 = 8.65 |
| Level 3 | 7,8,9 | (9.47+7.82+9.27)/3 = 8.85 |
| Uptake duration |  |  |
| Level 1 | 1,4,7 | (6.57+8.07+9.47)/3 = 8.03 |
| Level 2 | 2,5,8 | (7.56+9.03+7.82)/3 = 8.13 |
| Level 3 | 3,6,9 | (10.08+8.84+9.27)/3 = 9.40 |
| Time per projection |  |  |
| Level 1 | 1,6,8 | (6.57+8.84+7.82)/3 = 7.74 |
| Level 2 | 2,4,9 | (7.56+8.07+9.27)/3 = 8.30 |
| Level 3 | 3,5,7 | (10.08+9.03+9.47)/3 = 9.53 |
