# Supplementary material for: Optimization of Imaging Parameters for SPECT scans of [99mTc]TRODAT-1 Using Taguchi Analysis
Source: PLoS One. 2015 Mar 19;10(3):e0113817. doi: 10.1371/journal.pone.0113817 (PMC4366084; doi:10.1371/journal.pone.0113817)
Supplement: S3 Table — (DOCX) [file pone.0113817.s003.docx]

**Table S3. The raw data of the ten normal subjects and 15 PD patients to calculate the average S/O ratios and SNRs.**

For normal subjects

| **Setting** | ***y*_1_** | ***y*_2_** | ***y*_3_** | ***y*_4_** | ***y*_5_** |
| --- | --- | --- | --- | --- | --- |
| Convention | S:159.7  O:45.7 | S:270.2  O:72.5 | S:203.5  O:59.7 | S:186.2  O:52.1 | S:142.7  O:40.1 |
| Optimal | S:161.5  O:36.5 | S:182.4  O:46.2 | S:221.3  O:51.5 | S:175.8  O:41.4 | S:213.9  O:50.8 |

Group 3 data are adopted from Table 4.

For PD patients

| **Setting** | ***y*_1_** | ***y*_2_** | ***y*_3_** | ***y*_4_** | ***y*_5_** |
| --- | --- | --- | --- | --- | --- |
| Convention | S:121.7  O:49.3 | S:140.6  O:59.5 | S:146.4  O:76.5 | S:111.7  O:52.1 | S:84.2  O:40.6 |
| Optimal | S:96.2  O:46.4 | S:102.9  O:43.1 | S:133.1  O:51.7 | S:150.1  O:76.1 | S:114.2  O:50.8 |
| Original | S:101.5  O:46.9 | S:123.2  O:51.2 | S:87.1  O:47.6 | S:82.3  O:33.1 | S:97.4  O:42.5 |
